# Correction to “Single‐nucleus analysis reveals oxidative stress in Down syndrome basal forebrain neurons at birth”

**DOI:** 10.1002/alz.70777

**Published:** 2025-09-27

West NR, Arachchilage KH, Knaack S, et al. Single‐nucleus analysis reveals oxidative stress in Down syndrome basal forebrain neurons at birth. *Alzheimers Dement*. 2025;21(7):e70445. https://doi.org/10.1002/alz.70445. PMID: 40667939; PMCID: PMC12265022. The data presented in the current publication have been deposited in and are available from the dbGaP database under accession phs004098.v1.p1. Data can also be accessed through the INCLUDE Data Hub at the INCLUDE Data Coordinating Center. For comprehensive guides, documentation, and help in general, you may visit the INCLUDE DCC Help Center. The data can be interactively visualized at https://daifengwanglab.shinyapps.io/DS_basal/.

At the time of publication, the data were not publicly available. The data is now available.

We apologize for this error.

