# Supplementary material for: Single‐nucleus analysis reveals oxidative stress in Down syndrome basal forebrain neurons at birth
Source: Alzheimers Dement. 2025 Jul 16;21(7):e70445. doi: 10.1002/alz.70445 (PMC12265022; doi:10.1002/alz.70445)
Supplement: Supplementary file 8 — Supporting Information [file ALZ-21-e70445-s011.pdf]

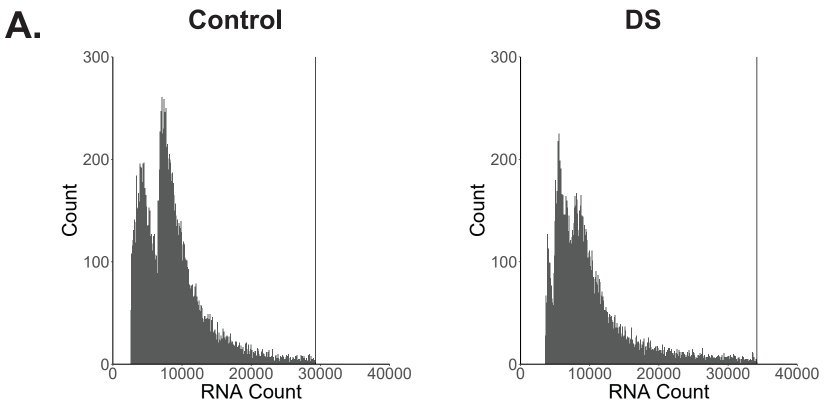

C.

	Control	DS
Doublets	1067	375
Low Quality	464	265
**Total Cells Removed**	1531	640

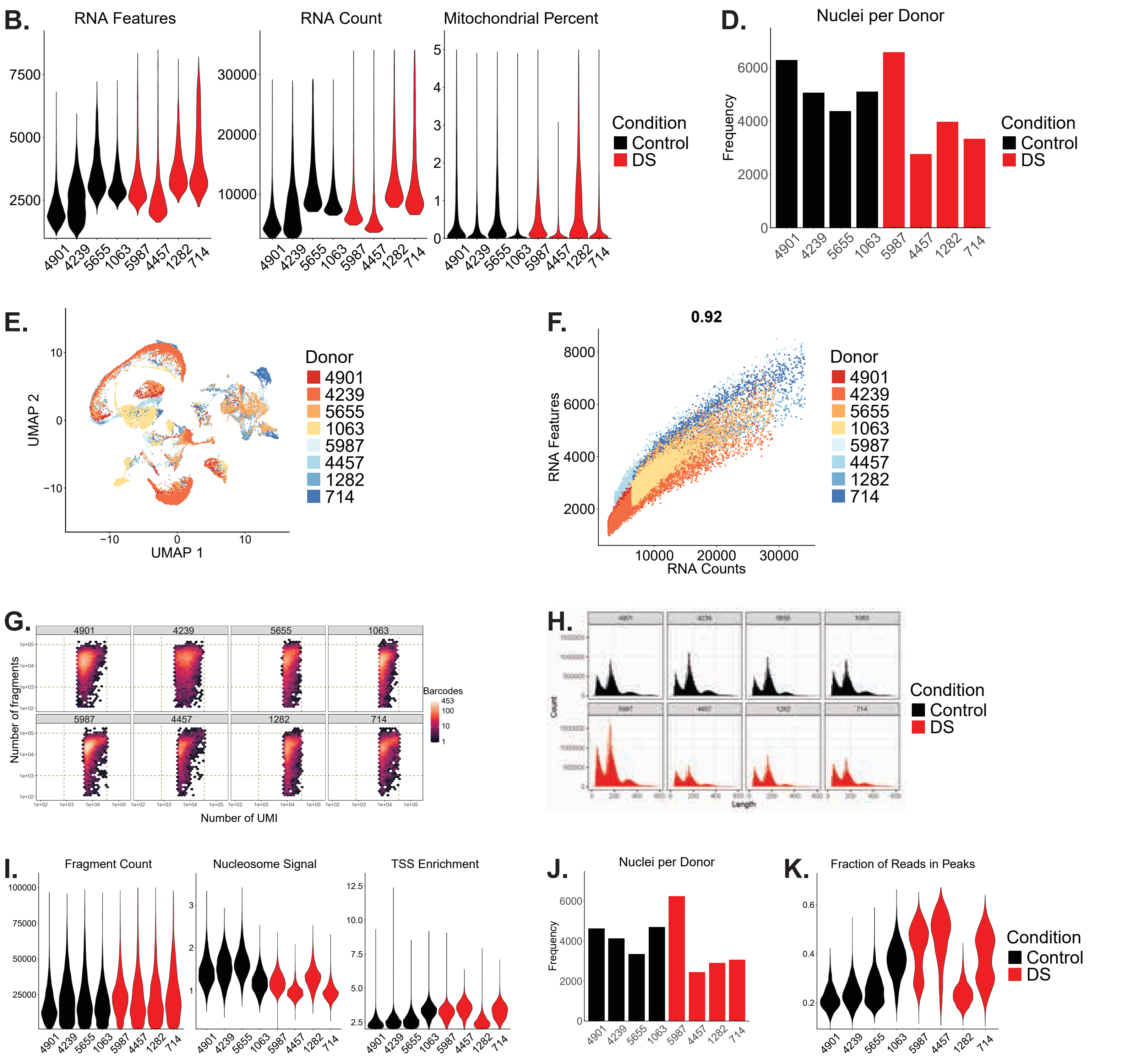

Supplement Figure 1. A) Quality control thresholds for control and DS samples. Nuclei were removed based on the following criteria: fewer than 200 expressed genes, ribosomal gene content exceeding 40%, mitochondrial gene content exceeding 5%, and a UMI count lower than 800 or higher than the upper threshold. The upper UMI thresholds were calculated individually for control and DS samples based on the lower and upper quartiles for each. The upper thresholds used were 29,284 for control and 34,160 for DS. B) RNA Features, RNA Count, and Mitochondrial Percent for each donor following QC. C) Additional nuclei removed per sample. Nuclei were removed if they were identified as a doublet in at least two out of three doublet detection methods, or if they were identified as 'low quality' and were not able to be confidently identified with known marker genes. D) Nuclei per donor after all QC. E) UMAP of all donors. F) There is a positive correlation (0.92) between the number of unique molecular identifiers (RNA Count) and the number of genes (RNA Features) per donor. G) QC thresholds for number of fragments and number of UMI per donor. H) Fragment length distribution showing the multi-nucleosomal positioning. I) Quality control thresholds for control and DS samples. Nuclei were removed based on the following criteria: number of fragments less than 1000 or greater than 100,000, a nucleosome fraction score > 4, and a TSS enrichment score < 2. J) Nuclei per donor after QC. K) Fraction of reads in peaks (FRiP) per barcode.
