# Supplementary material for: Single‐nucleus analysis reveals oxidative stress in Down syndrome basal forebrain neurons at birth
Source: Alzheimers Dement. 2025 Jul 16;21(7):e70445. doi: 10.1002/alz.70445 (PMC12265022; doi:10.1002/alz.70445)
Supplement: Supplementary file 9 — Supporting Information [file ALZ-21-e70445-s003.pdf]

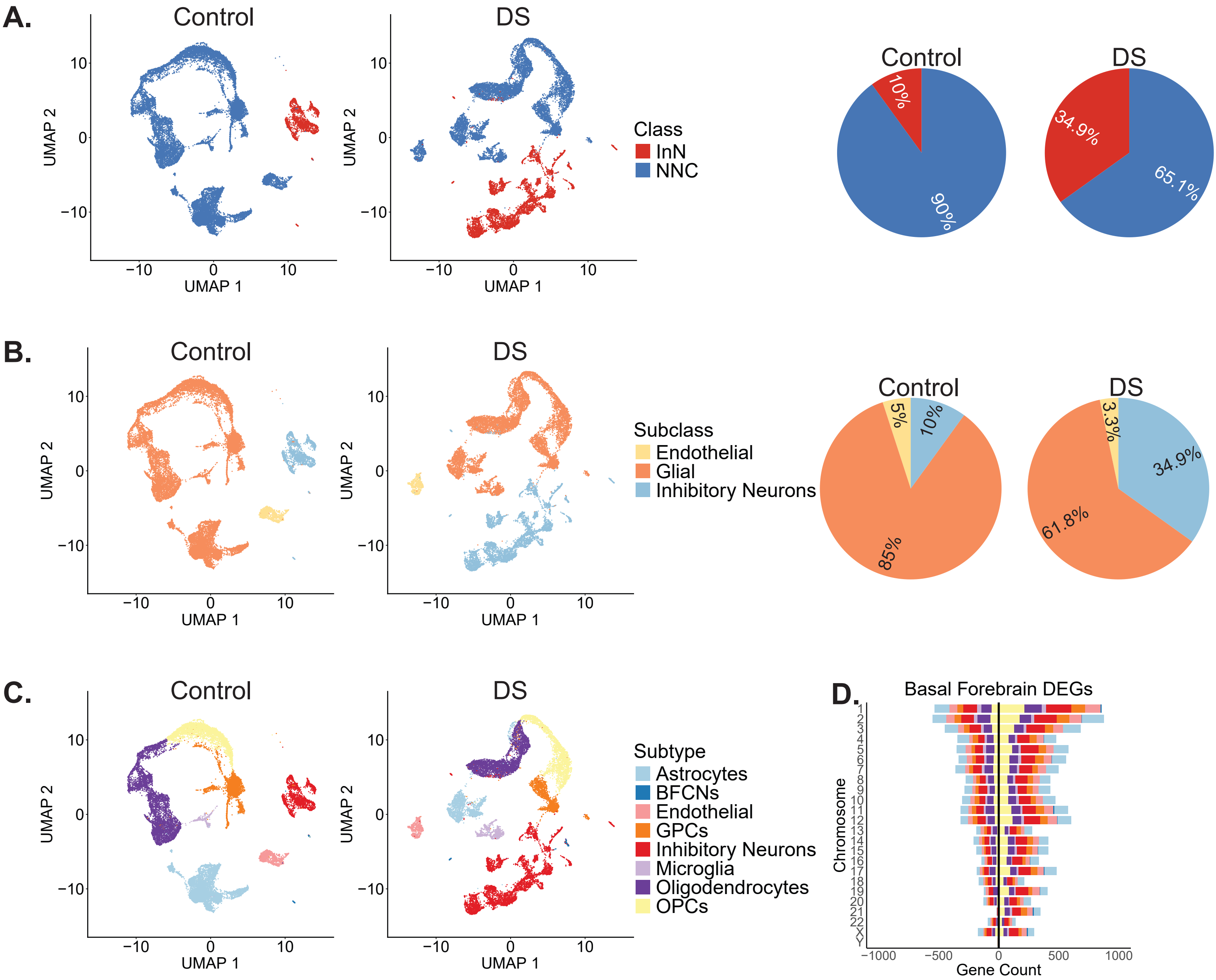

A.
Control
DS
UMAP 2
UMAP 1
Class
InN
NNC
Control
DS
10%
90%
34.9%
65.1%
B.
Control
DS
Subclass
Endothelial
Glial
Inhibitory Neurons
Control
DS
5%
10%
85%
3.3%
34.9%
61.8%
C.
Control
DS
Subtype
Astrocytes
BFCNs
Endothelial
GPCs
Inhibitory Neurons
Microglia
Oligodendrocytes
OPCs
D.
Basal Forebrain DEGs
Chromosome
Gene Count

**Supplement Figure 2.** A) UMAP of cell clusters by class split by control and DS. Percentages of cells in each class in control (N=20,826) and DS (N=16,641). No ExNs were identified in either sample. There is a higher percentage of InNs in DS and a higher percentage of NNCs in control. B) UMAP of cell clusters by subclass split by control and DS. Percentages of cells in each subclass in control (N=20,826) and DS (N=16,641). NNCs were identified as endothelial or glial cells and all InNs were annotated as inhibitory neurons at the subclass level. C) UMAP of cell clusters by subtype split by control and DS. Percentages of cells in each subclass in control (N=20,826) and DS (N=16,641). D) DEGs per chromosome by cell type. DEGs are distributed across the genome in all cell types.
