# Supplementary material for: Single‐nucleus analysis reveals oxidative stress in Down syndrome basal forebrain neurons at birth
Source: Alzheimers Dement. 2025 Jul 16;21(7):e70445. doi: 10.1002/alz.70445 (PMC12265022; doi:10.1002/alz.70445)
Supplement: Supplementary file 10 — Supporting Information [file ALZ-21-e70445-s002.pdf]

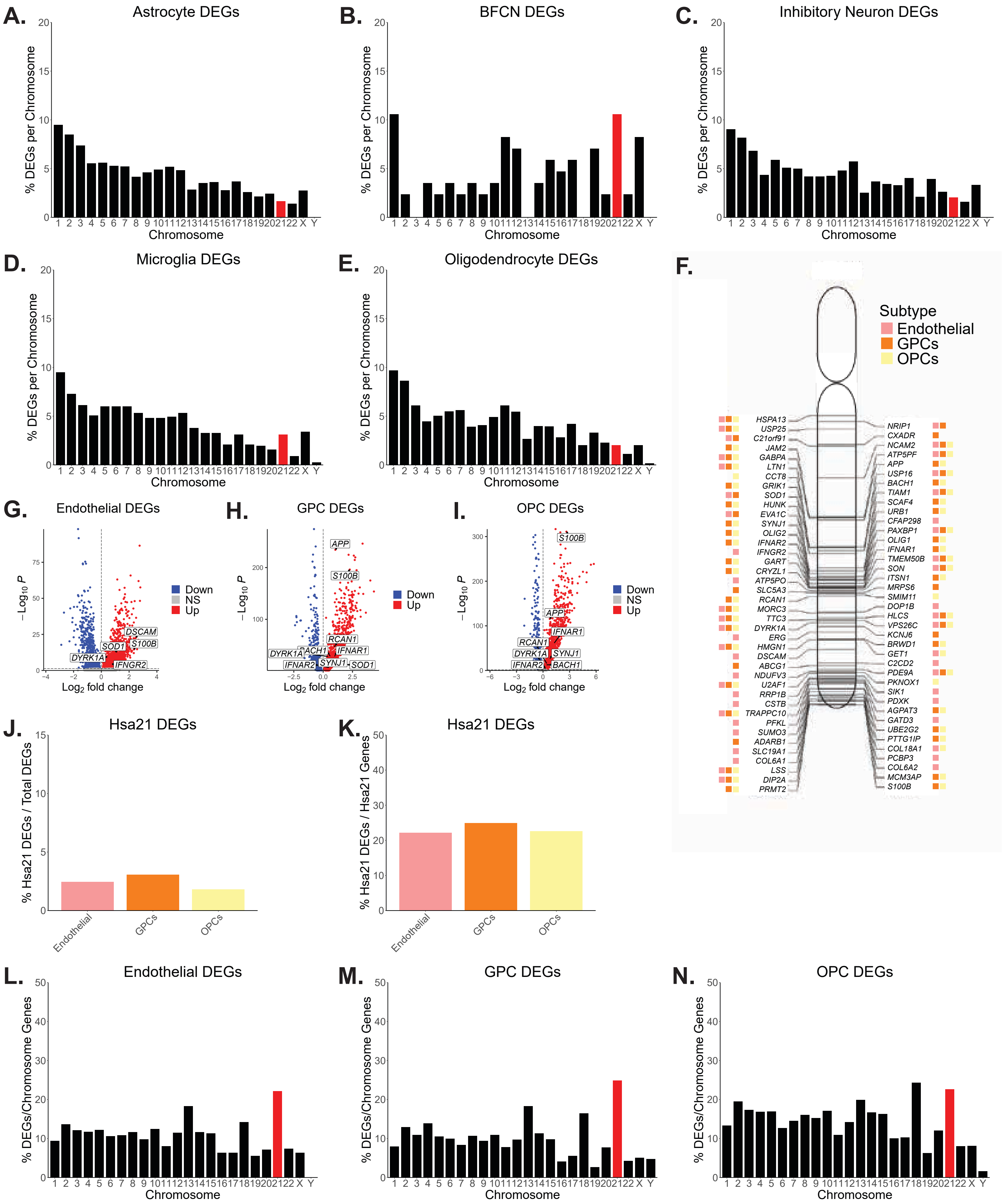
A.
Astrocyte DEGs
% DEGs per Chromosome
Chromosome
B.
BFCN DEGs
% DEGs per Chromosome
Chromosome
C.
Inhibitory Neuron DEGs
% DEGs per Chromosome
Chromosome
D.
Microglia DEGs
% DEGs per Chromosome
Chromosome
E.
Oligodendrocyte DEGs
% DEGs per Chromosome
Chromosome
F.
Subtype
Endothelial
GPCs
OPCs
HSPA13
USP25
C21orf91
JAM2
GABPA
LTN1
CCT8
GRIK1
SOD1
HUNK
EVA1C
SYNJ1
OLIG2
IFNAR2
IFNGR2
GART
CRYZL1
ATP5PO
SLC5A3
RCAN1
MORC3
TTC3
DYRK1A
ERG
HMGN1
DSCAM
ABCG1
NDUFV3
U2AF1
RRP1B
CSTB
TRAPPC10
PFKL
SUMO3
ADARB1
SLC19A1
COL6A1
LSS
DIP2A
PRMT2
NRIP1
CXADR
NCAM2
ATP5PF
APP
USP16
BACH1
TIAM1
SCAF4
URB1
CFAP298
PAXBP1
OLIG1
IFNAR1
TMEM50B
SON
ITSN1
MRPS6
SMIM11
DOP1B
HLCS
VPS26C
KCNJ6
BRWD1
GET1
C2CD2
PDE9A
PKNOX1
SIK1
PDXK
AGPAT3
GATD3
UBE2G2
PTTG1IP
COL18A1
PCBP3
COL6A2
MCM3AP
S100B
G.
Endothelial DEGs
H.
GPC DEGs
I.
OPC DEGs
$-Log_{10}$ P
$Log_2$ fold change
Down
NS
Up
DSCAM
SOD1
S100B
DYRK1A
IFNGR2
APP
S100B
RCAN1
DYRK1A
BACH1
IFNAR1
IFNAR2
SYNJ1
SOD1
APP
IFNAR1
RCAN1
DYRK1A
SYNJ1
IFNAR2
BACH1
J.
Hsa21 DEGs
% Hsa21 DEGs / Total DEGs
Endothelial
GPCs
OPCs
K.
Hsa21 DEGs
% Hsa21 DEGs / Hsa21 Genes
Endothelial
GPCs
OPCs
L.
Endothelial DEGs
% DEGs/Chromosome Genes
Chromosome
M.
GPC DEGs
% DEGs/Chromosome Genes
Chromosome
N.
OPC DEGs
% DEGs/Chromosome Genes
Chromosome

**Supplement Figure 3.** A) Percent of DS astrocyte DEGs on each chromosome. B) Percent of DS BFCN DEGs on each chromosome. C) Percent of DS inhibitory neuron DEGs on each chromosome. D) Percent of DS microglia DEGs on each chromosome. E) Percent of DS oligodendrocyte DEGs on each chromosome. F) Hsa21-encoded genes differentially expressed in endothelial cells, GPCs, and OPCs. Boxes next to each gene represent the cell types that gene is dysregulated in. G) Volcano plot of dysregulated genes in DS endothelial cells with Hsa21 genes of interest labeled. H) Volcano plot of dysregulated genes in DS GPCs with Hsa21 genes of interest labeled. I) Volcano plot of dysregulated genes in DS OPCs with Hsa21 genes of interest labeled. J) Percent of Hsa21 DEGs relative to total DEGs per cell type. K) Percent of Hsa21 DEGs normalized to total Hsa21 protein-coding genes per cell type. L) Percent of DEGs normalized to protein-coding genes per chromosome in DS endothelial cells with Hsa21 highlighted in red. M) Percent of DEGs normalized to protein-coding genes per chromosome in DS GPCs with Hsa21 highlighted in red. N) Percent of DEGs normalized to protein-coding genes per chromosome in DS OPCs with Hsa21 highlighted in red.
