# Supplementary material for: Single‐nucleus analysis reveals oxidative stress in Down syndrome basal forebrain neurons at birth
Source: Alzheimers Dement. 2025 Jul 16;21(7):e70445. doi: 10.1002/alz.70445 (PMC12265022; doi:10.1002/alz.70445)
Supplement: Supplementary file 11 — Supporting Information [file ALZ-21-e70445-s004.pdf]

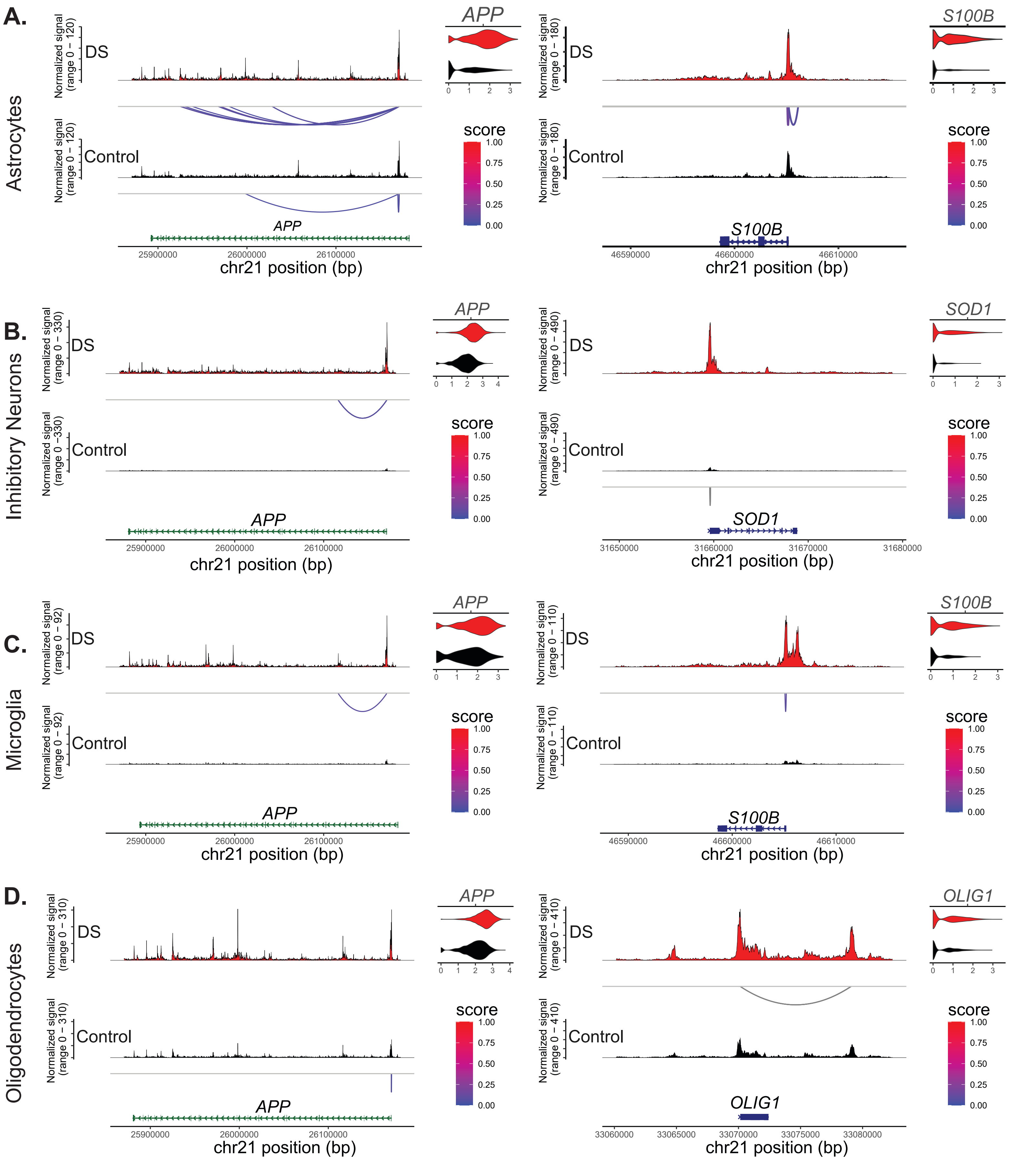

A.
Astrocytes
APP
S100B
DS
Control
Normalized signal (range 0 – 120)
Normalized signal (range 0 – 180)
score
1.00
0.75
0.50
0.25
0.00
25900000
26000000
26100000
46590000
46600000
46610000
chr21 position (bp)
B.
Inhibitory Neurons
SOD1
Normalized signal (range 0 – 330)
Normalized signal (range 0 – 490)
31650000
31660000
31670000
31680000
C.
Microglia
Normalized signal (range 0 – 92)
Normalized signal (range 0 – 110)
D.
Oligodendrocytes
OLIG1
Normalized signal (range 0 – 310)
Normalized signal (range 0 – 410)
33060000
33065000
33070000
33075000
33080000

Supplement Figure 4. A) Chromatin accessibility and peak-to-gene linkages for *APP* and *S100B* in control and DS astrocytes. B) Chromatin accessibility and peak-to-gene linkages for *APP* and *SOD1* in control and DS inhibitory neurons. C) Chromatin accessibility and peak-to-gene linkages for *APP* and *S100B* in control and DS microglia. D) Chromatin accessibility and peak-to-gene linkages for *APP* and *OLIG2* in control and DS oligodendrocytes. All of these Hsa21 genes have larger peaks around the TSS in DS, suggesting increased chromatin accessibility in the promoter region of each gene within the respective cell type.
