# Supplementary material for: Single‐nucleus analysis reveals oxidative stress in Down syndrome basal forebrain neurons at birth
Source: Alzheimers Dement. 2025 Jul 16;21(7):e70445. doi: 10.1002/alz.70445 (PMC12265022; doi:10.1002/alz.70445)
Supplement: Supplementary file 12 — Supporting Information [file ALZ-21-e70445-s005.pdf]

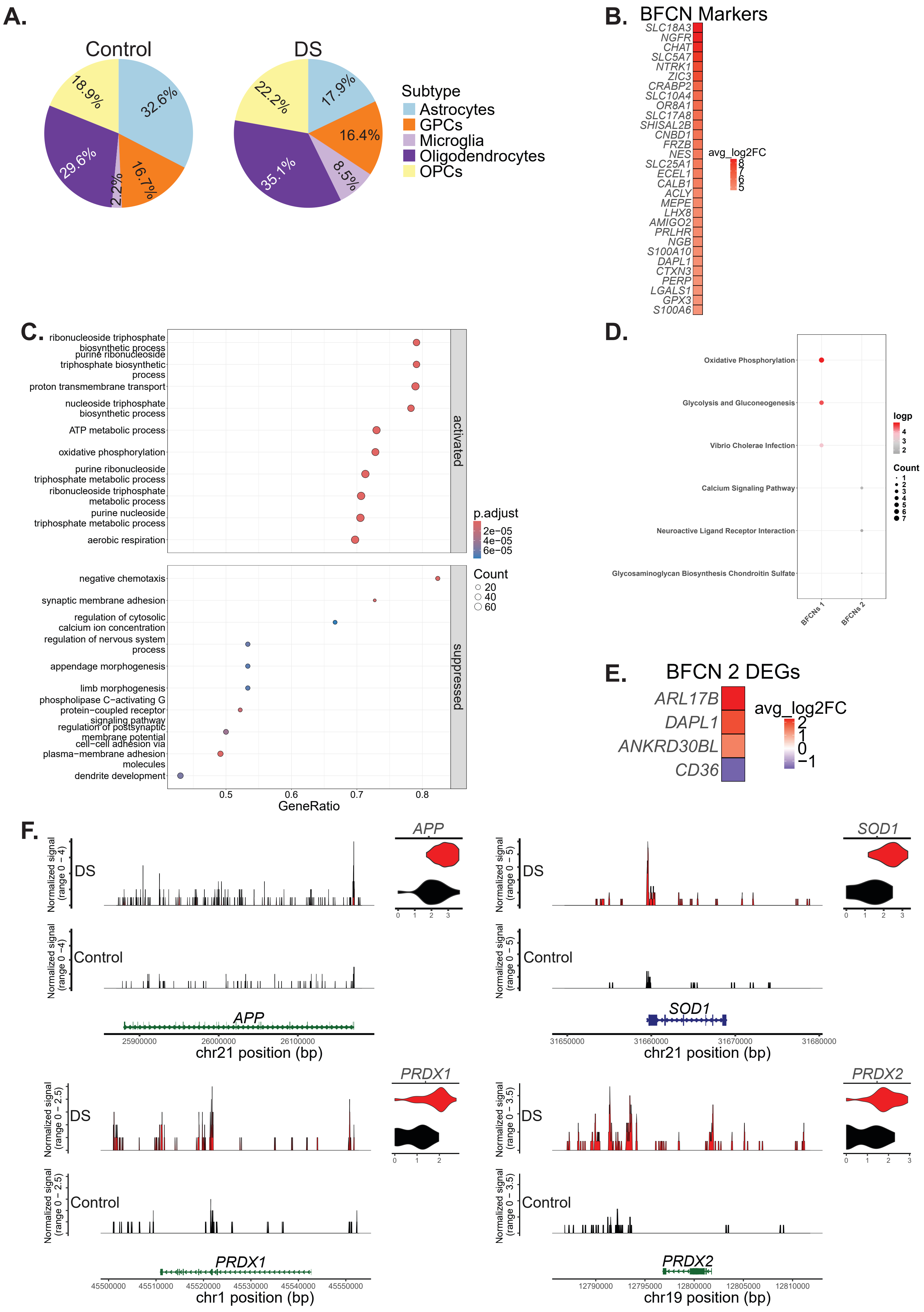
A.
Control
DS
Subtype
Astrocytes
GPCs
Microglia
Oligodendrocytes
OPCs
B. BFCN Markers
avg_log2FC
C.
activated
suppressed
p.adjust
Count
GeneRatio
D.
Oxidative Phosphorylation
Glycolysis and Gluconeogenesis
Vibrio Cholerae Infection
Calcium Signaling Pathway
Neuroactive Ligand Receptor Interaction
Glycosaminoglycan Biosynthesis Chondroitin Sulfate
logp
Count
BFCNs 1
BFCNs 2
E. BFCN 2 DEGs
ARL17B
DAPL1
ANKRD30BL
CD36
avg_log2FC
F.
APP
SOD1
PRDX1
PRDX2
DS
Control
chr21 position (bp)
chr1 position (bp)
chr19 position (bp)

Supplement Figure 5. A) Percentages of cell types in the glial subclass. There is a larger percentage of astrocytes in control and a larger percentage of microglia in DS. B) BFCN marker genes relative to inhibitory neurons. C) BFCNs 1 upregulated genes are enriched in metabolic processes whereas downregulated genes are enriched in cell adhesion and synapse formation relative to BFCNs 2. D) KEGG pathway analysis reveals genes enriched in oxidative phosphorylation and glycolysis/gluconeogenesis pathways are upregulated in BFCNs 1 compared to BFCNs 2. E) BFCNs 2 DEGs. F) Chromatin accessibility for Hsa21-encoded genes, *APP* and *SOD1*, and non-Hsa21-encoded antioxidant genes, *PRDX1* and *PRDX2*, in control and DS BFCNs.
